# Cardiac Non-myocyte Cells Show Enhanced Pharmacological Function Suggestive of Contractile Maturity in Stem Cell Derived Cardiomyocyte Microtissues

**DOI:** 10.1093/toxsci/kfw069

**Published:** 2016-04-28

**Authors:** Stephanie M. Ravenscroft, Amy Pointon, Awel W. Williams, Michael J. Cross, James E. Sidaway

**Affiliations:** *Department of Molecular and Clinical Pharmacology, MRC Centre for Drug Safety Science, Sherrington Building, the University of Liverpool, Ashton Street, L69 3GE, UK; ^†^Safety and ADME Translational Sciences, AstraZeneca R&D, Cambridge, CB4 0WG, UK

**Keywords:** cardiac microtissue, cardiotoxicity, hESC-CMs, inotropy, S100A1, contractility

## Abstract

The immature phenotype of stem cell derived cardiomyocytes is a significant barrier to their use in translational medicine and pre-clinical *in vitro* drug toxicity and pharmacological analysis. Here we have assessed the contribution of non-myocyte cells on the contractile function of co-cultured human embryonic stem cell derived cardiomyocytes (hESC-CMs) in spheroid microtissue format. Microtissues were formed using a scaffold free 96-well cell suspension method from hESC-CM cultured alone (CM microtissues) or in combination with human primary cardiac microvascular endothelial cells and cardiac fibroblasts (CMEF microtissues). Contractility was characterized with fluorescence and video-based edge detection. CMEF microtissues displayed greater Ca^2+ ^transient amplitudes, enhanced spontaneous contraction rate and remarkably enhanced contractile function in response to both positive and negative inotropic drugs, suggesting a more mature contractile phenotype than CM microtissues. In addition, for several drugs the enhanced contractile response was not apparent when endothelial cell or fibroblasts from a non-cardiac tissue were used as the ancillary cells. Further evidence of maturity for CMEF microtissues was shown with increased expression of genes that encode proteins critical in cardiac Ca^2+ ^handling (S100A1), sarcomere assembly (telethonin/TCAP) and β-adrenergic receptor signalling. Our data shows that compared with single cell-type cardiomyocyte *in vitro* models, CMEF microtissues are superior at predicting the inotropic effects of drugs, demonstrating the critical contribution of cardiac non-myocyte cells in mediating functional cardiotoxicity.

Drug associated cardiovascular toxicity causes severe morbidity (Schimmel *et al.*, 2004) and is a major reason for attrition during drug discovery and development (Abassi *et al.*, 2012; Albini *et al.*, 2010; Laverty *et al.*, 2011). It occurs at all stages of preclinical and clinical testing and may only be apparent during post-marketing several years after the cessation of treatment (Gwathmey *et al.*, 2009; Mellor, 2011). Drug induced cardiovascular toxicity can result in both functional effects such as arrhythmia and acute alteration of the contractile function (inotropy) of the heart, and morphological (structural) damage to the myocardium (Cross *et al.*, 2015; Laverty *et al.*, 2011). Evaluation of the potential for both types of cardiotoxicity by novel pharmacological compounds is essential for the discovery of safe drugs. Functional effects such as arrhythmias are screened for in cell lines over expressing key cardiac ion channels and cardiomyocyte contractility effects (positive and negative inotropy) are assessed in freshly isolated adult rat and canine cardiomyocytes (CMs) (Harmer *et al.*, 2012; Pollard *et al.*, 2010). Although the canine CMs can be cryopreserved (Abi-Gerges *et al.*, 2013), physiologically relevant human based *in vitro* models are currently lacking for risk assessment in man in an integrated system (Cross *et al.*, 2015). Furthermore, primary CMs from preclinical animal species are not amenable to plate-based high throughput screening (HTS) (Hescheler *et al.*, 1991; White *et al.*, 2004).

Recent advancements in stem cell biology, particularly the industrial scale production of differentiated stem cell derived CMs (Abassi *et al.*, 2012), offers an opportunity for developing a physiologically relevant model system that is amenable to drug discovery and for regenerative medicine. The utility of human embryonic stem cell (hESC) and induced pluripotent stem cell (iPSC) CMs for the prediction of drug-induced functional abnormalities is well documented (Abassi *et al.*, 2012; Caspi *et al.*, 2009; Guo *et al.*, 2011; Laposa, 2011; Rolletschek *et al.*, 2004; Zeevi-Levin *et al.*, 2012). However, the immature phenotype of stem cell derived CMs is considered a significant barrier to them fulfilling this potential in each of these areas (Yang *et al.*, 2014). When compared with adult CMs, stem cell derived CMs have a foetal gene expression profile, different myofiber subtypes, and disorganized sarcomeres (Guo *et al.*, 2011; Yang *et al.*, 2014). This immature phenotype also lacks T-tubules and is unable to give an inotropic response to β-adrenergic agonists and other positive inotropic compounds, which are seen *in vivo* (Pillekamp *et al.*, 2012; Pointon *et al.*, 2015).

Approximately 70% of the cells in the human heart are non-myocytes with cardiac fibroblasts and cardiac endothelial cells forming the majority of these non-myocyte cells (Brutsaert, 2003; Deb and Ubil, 2014). Cardiac fibroblasts serve a structural role by providing the extracellular matrix of the heart, mechanotransductive cues and paracrine factors that regulate cardiomyocyte maturation (Bowers *et al.*, 2010; Porter and Turner, 2009; Souders *et al.*, 2009), whilst cardiac endothelial cells form the myocardial microvasculature, which regulates the supply of oxygen and free fatty acids to the CMs (Aird, 2007). Cardiac endothelial cells also release paracrine factors that regulate cardiomyocyte metabolism, survival, and contractile function (Brutsaert, 2003; Narmoneva *et al.*, 2004).

Tissue engineering approaches offer great potential for developing 3D models of heart muscle for use in drug discovery and development and as cardiac tissue replacement in the clinic. These approaches include scaffold-free methods such as microtissues derived from hanging drops or in hydrogels and monolayer derived cell sheets or patches, and scaffold dependent methods (Hirt *et al.*, 2014). Mixtures of murine lung and heart derived microvascular endothelial cells were found to enhance neonatal cardiomyocyte survival and spatial organization in peptide hydrogels (Narmoneva *et al.*, 2004). Neonatal rat fibroblast and CMs co-cultured in agarose hydrogels resulted in prolonged action potential duration compared to cardiomyocyte cultures alone (Desroches *et al.*, 2012). The maturity of hESC-CMs was promoted by the presence of non-myocytes present in embryoid bodies (Kim *et al.*, 2010). Furthermore, the maturity of hESC-CMs was also enhanced by either human umbilical vein or hESC-derived endothelial cells, and fibroblast co-cultures in cell patches (Caspi *et al.*, 2007; Stevens *et al.*, 2009; Tulloch *et al.*, 2011)

Current *in vitro* approaches to analyse functional and structural cardiotoxicity are focussed on utilizing CMs alone (Cross *et al.*, 2015). Here for the first time, we have assessed the ability of adult primary cardiac microvascular endothelial cells and cardiac fibroblasts to induce contractile maturity in hESC-CM in scaffold-free microtissues. In particular, we have assessed the contractile phenotype in the context of pharmacological effects on cardiomyocyte contractility. Spheroid microtissues were formed from hESC-CM alone or in combination with human primary endothelial cells and fibroblasts obtained from either cardiac or noncardiac (dermal) sources. Compared to all other cell combinations, spheroid microtissues derived from all 3 cardiac cell types showed remarkably enhanced contractile function in response to inotropic drugs, which suggested a greater degree of cardiomyocyte maturity. Evidence of maturity was confirmed at the molecular and structural level. In addition, we show that cardiac specific fibroblasts and endothelial cells enhance and promote maturity through upregulation of the Ca^2+^-binding protein S100A1. We conclude that noncardiomyocyte cells are critical for promoting an authentic cardiac phenotype in complex *in vitro* models and that such models can be used for more accurate drug safety screening and drug development.

## MATERIALS AND METHODS

### Preparation of cardiac microtissues

hESC-CMs (Cytiva) were obtained from General Electric Healthcare (Hertfordshire, UK). Human induced pluripotent stem cell CMs (hiPS-CMs) were obtained from Cellular Dynamics International (Madison, Wisconsin). Primary human cardiac microvascular endothelial cells (hCMECs), primary human dermal microvascular endothelial cells (hDMECs), primary human coronary artery endothelial cells), primary human cardiac fibroblasts (hCFs), and primary normal human dermal fibroblasts (NhDFs) were obtained from PromoCell (Heidelberg, Germany). All primary cells were subcultured prior to microtissue formation according to supplier’s instructions. All primary cells were detached with pre-warmed Accutase (Sigma, A6964) for 5 min at 37°C, 5% CO_2_, centrifuged for 3 min at 1200 × *g* before re-suspension in endothelial basal medium MV2 (PromoCell, C-22221) (supplemented with 5% foetal calf serum (FCS), epidermal growth factor (EGF; 5 ng ml^−1^), basic fibroblast growth factor (bFGF; 10 ng ml^−1^), insulin-like growth factor 1 (IGF-1; 20 ng ml^−1^), vascular endothelial growth factor-A (VEGF-A; 0.5 ng ml^−1^), Ascorbic Acid (1 μg ml^−1^) and Hydrocortisone (0.2 μg ml^−1^). Cell suspensions were counted, diluted and stored at 37°C, 5% CO_2_ for up to one hour while hESC-CM cell suspensions were prepared. A vial of Cytiva CMs was thawed and suspended in RPMI 1640 media (Life technologies 61870044) containing b27 supplement (Life technologies 17504) (Pointon *et al.*, 2013). CMEF/DMEF microtissues: cell suspensions were combined together to give a concentration of 500 cells per 100 μl comprising 4 parts hESC-CM, 2 parts hCF/NhDF, and 1 part hCMEC/hDMEC in microtissue media comprising 1 part RPMI (+b27 supplement) to 1 part Endothelial MV2 (+supplements). CME/CMF microtissues: cell suspensions were mixed together to give a concentration of 500 cells per 100 μl comprising 2 parts hESC-CM and one part hCF/hCMEC in microtissue media. CM microtissues: hESC-CMs suspension was diluted to 1000 cells per well in RPMI (+b27 supplement) media. In microtissues containing hiPS-CMs, hESC-CMs were replaced with hiPS-CMs. hiPS-CMs were prepared in iCell thawing media as previously described in Pointon *et al.* (2015). After 48 h culture media was refreshed with iCell CM maintenance media. Suspensions were subsequently seeded into round bottom ultra- low adhesion 96-well plates (Corning Costar, CLS7007).

All microtissue plates were incubated at 37°C, 5% CO_2_ for 14 days with media refreshed every 3–4 days. Experiments were conducted on microtissues following 14–28 days in culture.

### Immunofluorescence

Microtissues were washed in D-phosphate buffered saline (PBS) until no media remained and subsequently fixed in 4% (w/v) para-formaldehyde for 1 h at 4°C, followed by 6 washes in D-PBS and stored at 4 °C until processing. Microtissues were permeabilized with 0.5% (v/v) triton X-100/D-PBS overnight at 4°C then blocked in 3% (w/v) BSA/TBST block (with 0.1% (v/v) Triton X-100) for 2 h at room temperature (RT). Primary antibody was diluted as required in 1% (w/v) BSA/TBST (with 0.1% (v/v) Triton X-100), block reagent was removed and primary antibody added overnight at 4 °C. Microtissues were washed three times with TBST (with 0.1% (v/v) Triton X-100) at RT, each time incubated for 1 h. Secondary antibody was diluted 1:1000 in 1% (w/v) BSA/TBST (with 0.1% (v/v) Triton X-100) and added to the microtissues overnight at 4 °C. Microtissues were washed for 1 h with TBST (with 0.1% (v/v) Triton X-100) at RT and stained with Hoechst 33342 (2 μg/ml) in TBST (with 0.1% (v/v) Triton X-100) for 1 h at RT. Microtissues were washed briefly in TBST (with 0.1% (v/v) Triton X-100) before mounting onto microscope slides with ProLong Gold. Microtissues were imaged using a Zeiss AxioObserver inverted fluorescence microscope with Apotome 2, using Plan-Apochromat 20×/0.8 NA objective. Images were acquired using Zeiss Zen software. In order to allow direct comparisons between different microtissues, during image acquisition each microtissue was processed in a blinded manner and equal exposure was applied for each marker.

### Gene expression analysis

Adult left ventricle, foetal heart, and smooth muscle total RNA were purchased from U.S. Biologic (Memphis, USA). Adult left ventricle heart total RNA was obtained from one 21-year old normal male donor with no reported concomitant disease. hESC-CM total RNA was obtained from hESC-CMs cultured as described in Pointon *et al.* (2013). RNA was harvested following 72 h in culture as per the recommended manufacturer instructions. All primary cells were detached with prewarmed Accutase (Sigma, A6964) for 5 min at 37°C, 5% CO_2_, centrifuged for 3 min at 1200 ×g before lysis in RLT buffer (Qiagen) and stored at −80 °C until processing. Microtissues were pooled and transferred into a falcon tube, centrifuged at 1200 ×g for 2 mins and re-suspended in prewarmed PBS. Microtissues were re-centrifuged at 1200 ×g for 2 min and lysed in RLT buffer (Qiagen). Total RNA was extracted using an RNeasy Mini Kit (Qiagen). A NanoDrop spectrophotometer (NanoDrop Technologies) was used to quantify RNA samples.

RNA samples were diluted to 20 ng and reverse transcribed using the SuperScript III first-strand synthesis supermix (Life technologies). cDNA samples were preamplified using the TaqMan PreAmp Master mix kit with TaqMan Probes (Supplementary Table S1), 10 cycles and final stock diluted 1:250. qRT-PCR was performed using 2.5 μl of preamplified cDNA and TaqMan Gene Expression master mix. qRT-PCR reactions were performed in a 7900 HT Fast Real-Time PCR System (Applied Biosystems, Foster City, California), 2.5 μl of preamplified cDNA was used with TaqMan Gene Expression probes (Supplementary Table S1) and master mix to monitor amplification under standard cycling conditions. GAPDH was used as an endogenous control. Relative quantification of gene expression was performed using the ΔΔCt relative quantitation method. The mean Ct value was calculated for each gene and normalized to the endogenous control, fold change relative to hESC-CM monolayer was determined and data expressed as a percentage of adult ventricle heart expression levels.

### Solutions and compounds

Compounds were obtained from Sigma-Aldrich (Dorset, UK) or the AstraZeneca compound collection. Compounds were initially formulated in DMSO as a 1000× stock solution and subsequently serially diluted using DMSO. The DMSO stock solutions were diluted 1000× into the cardiac microtissue media to give the final test concentrations. The final vehicle concentration was 0.1% (v/v) DMSO. Where compounds were insoluble in DMSO, PBS, (Sigma) was used. Caffeine was formulated in microtissue media at 10 mM.

### Video-based edge detection of contractions

Microtissues were transferred onto 0.1% (w/v) gelatin-coated 13 mm plastic coverslips (Thermanox) within a 12-well plate and incubated at 37°C, 5% CO_2_ for at least 1 h before mounting onto the IonOptix contractility system. Contractile properties of microtissues were assessed using a video-based edge detection method (IonOptix, Dublin, Ireland) (Harmer *et al.*, 2012). A gelatin-attached microtissue was placed in a perfusion chamber (FHC Inc., Bowdoinham, Maine) mounted on the stage of an inverted Nikon TE200 microscope (Nikon UK, Surrey, UK). The microscope stage (Prior Scientific Instruments, Cambridge, UK) was motorized and controlled remotely. Microtissues were continuously perfused from a gravity fed system at 2 ml/min with microtissue media heated to 37 ± 1 °C using a line heater (Cell MicroControls, Norfolk, Virginia). The microtissues were field stimulated with suprathreshold voltage at a pacing frequency of 1 or 1.5 Hz, with a bipolar pulse of 6 ms duration, using a pair of platinum wires placed on opposite sides of the chamber connected to a MyoPacer EP stimulator (IonOptix). Microtissues were imaged at 240 Hz using an IonOptix Myocam-S CCD camera. Digitized images were displayed within the IonWizard acquisition software (IonOptix). The edge of the microtissue of interest was aligned parallel to the video raster line, by means of a semi-automated dovecote prism optical system that could be controlled remotely (Cairn Research, Faversham, UK). Optical intensity data were collected representing dark and light bands at the edge of the microtissue of interest. The IonWizard software analyzes the periodicity in the optical density by means of a fast Fourier transform algorithm. The output of this analysis is a direct real-time measurement of microtissue contraction.

Each compound response was measured from separate randomly selected microtissues on triplicate independent occasions, only one microtissue was assessed at a time. After microtissues were added to the microscope stage, perfusion with microtissue media (with 0.1% (v/v) DMSO as the vehicle control) was immediately started. At this time field stimulation was also started, and microtissues were left to acclimatize for approximately 2 min at approximately 37 °C, after which at least 30 s of basal contractions were recorded for each microtissue. Test compounds were applied in a cumulative manner with each concentration applied for 250 s or until steady-state was achieved. Every 3–4 days, 10 μM verapamil was applied as a positive control, additionally beat rate was assessed at the start and end of the experimental time frame (14–28 days) to ensure consistency. Data from any microtissue batches that either did not elicit a response to 10 μM verapamil and/or varied in beat rate >10% over the time period were rejected.

Initial analysis was performed using the IonWizard software. For each test condition, data for 10–15 contractions were averaged, using the stimulation time as common reference point, to give a single representative monotonic contractility transient. This transient was analysed using the ‘monotonic transient analysis’ function of the IonWizard software. From this analysis, peak height, which indicates the percentage of peak contraction relative to the resting length was used to quantify microtissue dynamics. Concentration-effect curves were plotted for each compound and expressed relative to vehicle (0.1% (v/v) DMSO).

### Ca^2+^ transient measurements

Microtissues were loaded with 0.2 µmol/l Fura-2-acetoxymethyl ester (Life technologies, Paisley UK), a calcium-sensitive, ratiometric fluorescence dye, for 30 min at RT and kept in the dark. The same experimental design used for performing contractility recordings was applied to carry out intracellular Ca^2+^ measurements (Pointon *et al.*, 2015). The IonOptix system was used to conduct the experiments, including the Xenon arc lamp, hyperswitch and myopacer for microtissue stimulation and fluorescence excitation, myocam-S for the measurement of edge contraction, and a fluorescence system interface to integrate the different components (IonOptix Ltd). The Xenon arc lamp and fluorescence hyperswitch containing a galvanized mirror were used to alternate between wavelengths of 340 and 380 nm at a high frequency. Ca^2+^ fluorescence was recorded at 510 nm. Ca^2+ ^traces represent the ratio of Ca^2+ ^bound: Ca^2+ ^free Fura-2-acetoxymethyl ester dye, and hence changes in free intracellular Ca^2+^. Simultaneous contraction and Ca^2+ ^recordings for microtissues were taken while perfusing with their standard cell culture media containing DMSO 0.1% (v/v) as the vehicle control when appropriate. A transition period of 250 s was conducted between test concentrations, allowing any compound induced effects to reach a steady state. Analysis was performed using the IonWizard software as for contractility measurements.

### S100A1 knockdown

Microtissues were transfected with siRNA using Lipofectamine RNAiMAX transfection reagent. S100A1 siRNA and negative control scrambled siRNA was purchased from Life Technologies (Paisley, UK) Both siRNA probes and Lipofectamine RNAiMAX transfection reagent were diluted in OptiMEM to give a final concentration of 0.1% (v/v) for the later. The diluted Lipofectamine RNAiMAX and siRNA components were combined, mixed well by gentle pipetting and incubated for 5 min at RT. Cell culture media was removed from the microtissues and replaced with 100 μl of transfection solution. The plates were swirled gently to ensure even distribution of the siRNA. Microtissues were incubated with the transfection solution at 37 °C, 5% CO_2_ for 24 h prior to use.

### Data analysis

Concentration-effect curves were plotted for each compound and expressed relative to vehicle ± SD. The molar concentration of test compound producing 50% inhibition (IC_50_) or 50% response (EC_50_), was calculated using GraphPad Prism (La Jolla, California) to fit data to a 4-parameter nonlinear regression curve. Each IC_50_/EC_50_ was generated in triplicate for each compound and geometric means were subsequently calculated. F-tests to compare the concentration response curves respectively were performed using GraphPad Prism (La Jolla, Califorrnia). *T*-tests and ANOVA to compare the conditions were performed using GraphPad Prism (La Jolla, Califorrnia).

## RESULTS

### Development of Cardiac Microtissues

**FIG. 1:**
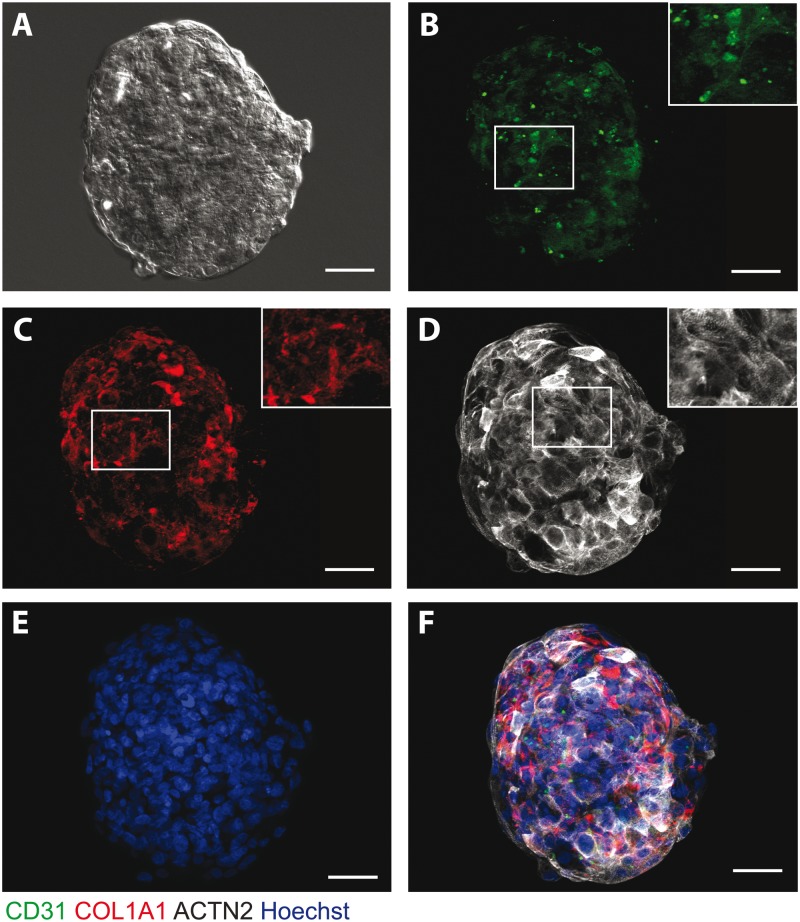
Structural characterization of CMEF microtissues. **A,** DIC image of a representative CMEF microtissue. Immunofluorescent staining of CMEF microtissues for **(B)** endothelial marker, CD31 (green); **(C)** fibroblast marker, collagen I (COL1A1) (red); **(D)** cardiac striation marker, ACTN2 (white); **(E)** nuclei stain, hoechst (blue) and **(F)** Merge of CD31 (green), COL1A1 (red), ACTN2 (white) and hoechst (blue). Scale bar represents 50 μm.

**FIG. 2:**
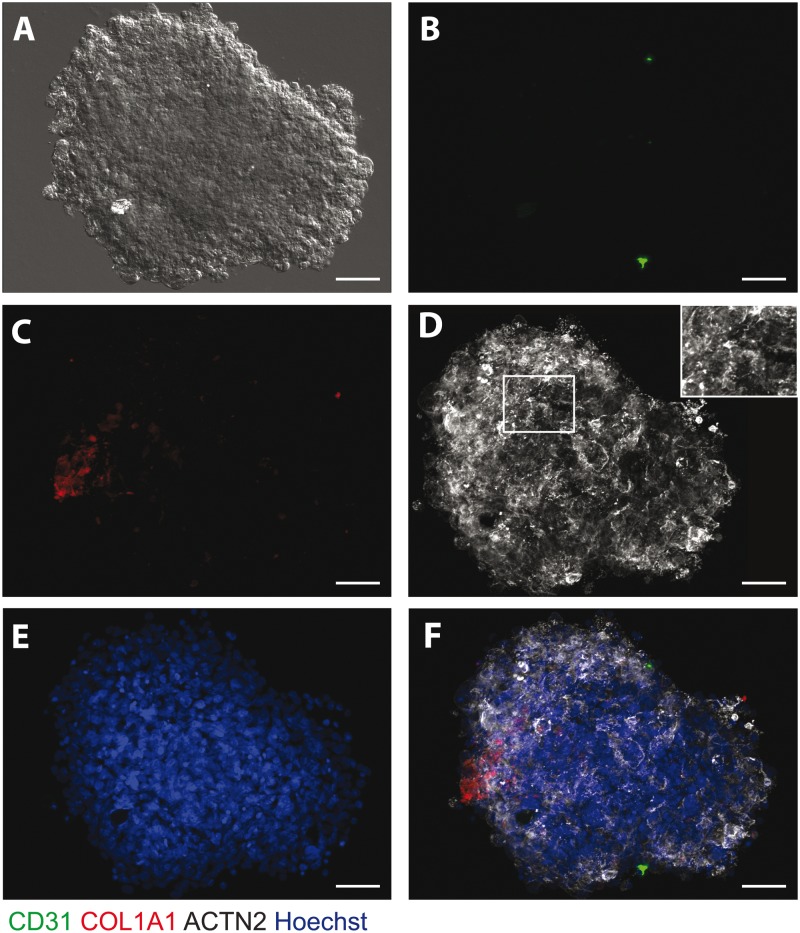
Structural characterization of CM microtissues. **A,** DIC image of a representative CM microtissue. Immunofluorescent staining of CM microtissues for **(B)** endothelial marker, CD31 (green); **(C)** fibroblast marker, collagen I (COL1A1) (red); **(D)** cardiac striation marker, ACTN2 (white); **(E)** nuclei stain, hoechst (blue) and **(F)** Merge of CD31 (green), COL1A1 (red), ACTN2 (white) and hoechst (blue). Scale bar represents 50 μm.

hESC-CMs were cultured either alone (CM microtissues) or with hCMECs and hCFs (CMEF microtissues) in 96-well round-bottomed ultra-low adhesion plates. Following two weeks in culture, microtissues with uniform size and shape, measuring 200 μm in diameter, with equal light exposure were formed (Figs. 1A and 2A). Immunofluorescence imaging of specific cardiac cell types: cardiomyocyte (alpha actinin, ACTN2), endothelial (CD31), and fibroblast markers (collagen I, COL1A1) was utilized to determine the morphology and cellular architecture of the microtissues. The presence of hCMECs (CD31) (Figure 1B) and hCFs (collagen 1) (Figure 1C) were confirmed in the CMEF microtissue and located in close proximity to the hESC-CMs, displaying even cell type distribution throughout. Staining of α-actinin highlights hESC-CMs within the CMEF and CM microtissues. Differences in cardiac striations between the CM and CMEF microtissues were revealed following imaging of α-actinin with the latter displaying striations with enhanced organization and elongation (Figs. 1D and 2D).

### CMEF Microtissues Display Improved Contractile Properties

**FIG. 3:**
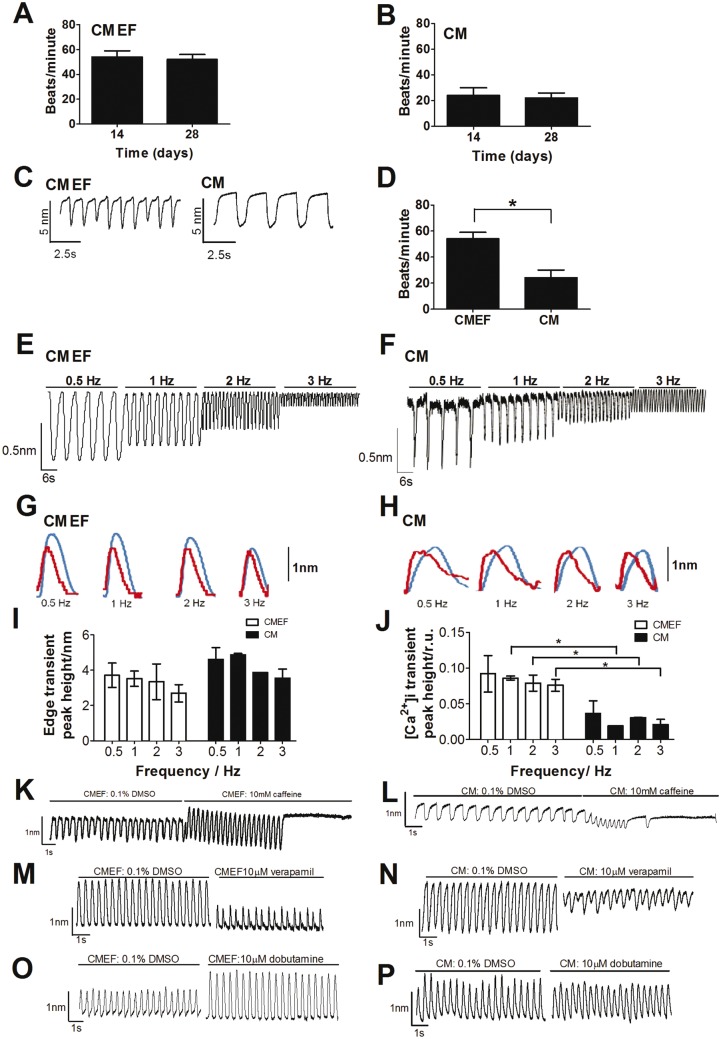
Functional characterization of CMEF and CM microtissues. Contractile properties (edge) and Ca^2+ ^kinetics were assessed using a video-based edge detection method (IonOptix, Dublin, Ireland). Microtissues were formed and incubated for 14 days prior to measurment of the spontaneous beat rate in both CMEF **(A)** and CM microtissues **(B)** at the start (day 14) and end (day 28) of the experimental period. Plates were incubated at 37 °C, 5% CO_2_ for 14 days with media refreshed every 3–4 days. Experiments were conducted on microtissues following 14–28 days in culture. Representative trace showing contraction (edge) kinetics in spontaneous contracting CMEF and CM microtissues **(C)**, the number of contractions over a period of time were quantified **(D)** (n > 3, mean ± SD). Representative recordings of CMEF **(E)** and CM microtissue **(F)** contractions following field stimulation at 0.5, 1, 2, and 3 Hz. Representative traces following measurement of Ca^2+ ^(red trace) and contraction transients (edge) (blue trace) in **(G)** CMEF and **(H)** CM microtissues following field stimulation at 0.5, 1, 2, 3 Hz with 7V for 6 ms. Summary of the frequency-response relationship for contraction (edge) **(I)** and [Ca^2+^]_i_ **(J)** at 0.5, 1, 2, and 3 Hz, n = 3, (±) SD in both CMEF (open bars) and CM microtissues (solid bars) *.*A representative experiment showing the effect of 10 mM caffeine on contraction transients elicited at 1.5 Hz stimulation in CMEF **(K)** and CM **(L)** microtissues.20 s of vehicle control (0.1% DMSO) contraction transients (left side) and >10 s of 10mM caffeine contraction transients (right side) are shown. Representative contractility transients elicited at 1.5 Hz stimulation in CMEF **(M)** and CM **(N)** microtissues following 10 μM verapamil. 20 s of basal contraction transients (left side) and 20 s of 10 μM verapamil contraction transients (right side) are shown. . Representative contractility transients elicited at 1.5 Hz stimulation in CMEF **(O)** and CM **(P)** microtissues following 10 μM dobutamine. 20 s of basal contraction transients (left side) and 20s of 10 μM dobutamine contraction transients (right side) are shown. *P-value < .01. (Full color version available online.)

To determine the contraction and relaxation kinetics of the microtissues, we used the IonOptix cell geometry measurement system and IonWizard software. This allowed measurement of movement of microtissues in real time at 37 ± 1°C, with a continuous flow of cell culture media, while spontaneously beating and following field stimulation at 0.5, 1, 2, and 3 Hz (i.e. equivalent to 30, 60, 120, and 180 beats/min). Under these conditions both microtissues displayed typical spontaneous synchronized contractility transients, with a spontaneous beat rate of 54 ± 5 and 24 ± 6 beats/min in CMEF and CM microtissues, respectively (mean ± SD, n = 3) (Figs. 3C and D); P value < .01. This spontaneous beat rate for each tissue remained constant over the period of study (14–28 days) confirming the viability of tissues (Figs. 3A and B). Furthermore, both microtissues were able to maintain synchronized, typical contraction transients following field stimulation (7 V for 6 ms) at 0.5, 1, 2, and 3 Hz (Figs. 3E and F), resulting in a mean beat rate of 29± 1.1, 59 ± 1.5, 90 ± 1.5, and 123 ± 8.5 beats/min, respectively in CMEF microtissues and 21± 3, 58 ± 4.1, 91 ± 3.2, and 119 ± 5.5 beats/min, respectively in CM microtissues (n = 3 ± SD). As changes in [Ca^2+^]_i_ are intrinsically linked to changes in cardiomyocyte contraction, we investigated the relationship between these parameters in both microtissues. In field-stimulated (0.5, 1, 2, and 3 Hz) CM and CMEF microtissues, the rise in [Ca^2+^]_i_ occurred before the initiation of contraction (Figs. 3G and H). Both microtissues showed a frequency–response relationship: as the frequency increased, the amplitude of the [Ca^2+^]_i_ and contraction transients decreased (n = 3) (Figs. 3I and J). However, the CMEF microtissue produced Ca^2+ ^transients of that were of higher amplitude at 1, 2, and 3 Hz. In order to assess the functionality of Ca^2+ ^handling, microtissues were acutely exposed to caffeine (10 mM) through a short timescale exposure in the perfused media and contraction transients monitored during spontaneously beating conditions. CMEF microtissues produced an immediate transient increase in contraction number and amplitude, followed by cessation of contraction (Figure 3K). Whereas in CM microtissues; contractions gradually decreased in both number and amplitude prior to cessation of contraction (Figure 3L).

Due to these observed differences in Ca^2+ ^handling, the pharmacological response following 1.5 Hz field stimulation to a known *in vivo* negative (verapamil) and positive (dobutamine) inotrope was assessed to determine the suitability of these microtissues for the identification and investigation of changes in cardiac contractility. Contractility transients were recorded following vehicle control (0.1% (v/v) DMSO) and each compound on three independent occasions (n = 3). To take into account differences in basal contraction kinetics between microtissues, all data was normalized to the vehicle response (0.1% DMSO (v/v)) in each microtissue. Consequently any differences observed are a direct result of compound exposure. In both CMEF and CM microtissues, 10 μM verapamil reduced the contractility transients (Figs. 3M and N), whereas following 10 μM dobutamine the CMEF microtissue produced a positive inotropic response with an increase in contractile amplitude, but no effect was observed in the CM microtissue (Figs. 3O and P). This data demonstrates the potential utility of microtissues for the detection and investigation of cardiac contractility *in vitro*.

The potential of cardiac microtissues to detect a pharmacologically diverse panel of 12 inotropic compounds with *in vivo* changes in cardiac contractility (5 negative and 7 positive inotropes) and 5 noninotropic compounds without *in vivo* changes in cardiac contractility were assessed following field stimulation at 1.5 Hz. Cumulative concentration response curves and corresponding IC_50_/EC_50_ values were obtained for each compound in both CMEF and CM microtissues. Overall, all positive inotropes tested were detected with an increase in amplitude of contraction in CMEF microtissues. In comparison, in CM microtissues 4 out of 7 positive inotropes were detected; however, the direction of response was the reverse to that observed *in vivo* for all except epinephrine (Table 1). For example, the Na+/K+ ATPase membrane pump inhibitor, digoxin, increased the amplitude of contractility transients in CMEF microtissues, producing an EC_50_ value of 3.54 μM. In contrast, in CM microtissues a decrease in the amplitude of contractility transients was observed, resulting in an IC_50_ value of 0.4 μM. Additionally, following exposure to 5 negative inotropes, all were correctly detected in CMEF, but only 3 (diltiazem, doxorubicin, verapamil) were detected in the CM microtissue (Table 1). For example, the β1-adrenergic receptor antagonist, atenolol, resulted in IC_50_ values of 0.94 μM and above 100 μM in CMEF and CM microtissues respectively. This data indicates the superior utility of CMEF microtissues compared to CM microtissues with respect to *in vitro* to *in vivo* pharmacological correlation and relevance.

**TABLE 1:** Contractile Responses of CMEF and CM Microtissues to Known Pharmacological Agents

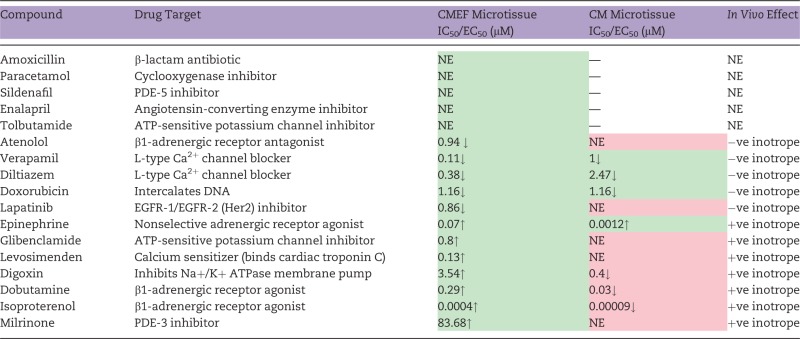

Contractile response of CMEF and CM microtissues to a panel of 12 pharmacologically diverse inotropic compounds. Drug targets and known *in vivo* effects are shown. IC_50_/EC_50_ shown (n = 3). Direction of inotropic response highlighted with arrows (↑ increase in inotropy, ↓decrease in inotropy). Correct prediction compared to *in vivo* effect highlighted in green, incorrect prediction compared with *in vivo* effect highlighted in red.

### Contractile Pharmacology Is Dependent upon a Cardiac tri-Culture System

**FIG. 4:**
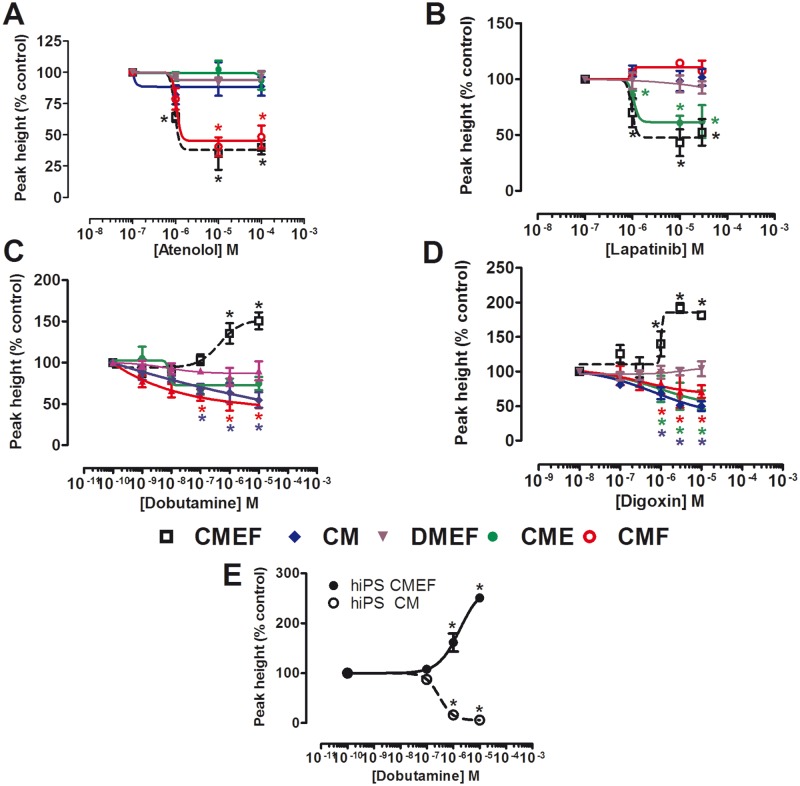
The contractile response of cardiac microtissue models to known inotropic compounds. Concentration-effect curves of CMEF, CM, DMEF, CME, and CMF microtissues to **(A)** atenolol, **(B)** lapatinib, **(C)** dobutamine, and **(D)** digoxin. **E,** concentration-effect curves of microtissues containing hiPS-CMs alone or in combination with hCMECs and hCF response to dobutamine. Data expressed relative to vehicle (0.1% (v/v) DMSO) (n = 3 ± SD). *P-value < .05.

In order to further explore these differences in responses between CMEF and CM microtissues, three additional microtissues were evaluated. The first additional microtissue contained hESC-CMs and hCMECs (CME microtissue), the second contained hESC-CMs and hCF (CMF microtissue) and the third microtissue contained both endothelial and fibroblasts from a dermal vascular bed with hESC-CMs (DMEF microtissue; Supplementary Figure S1). Two negative (atenolol, lapatinib) and two positive (digoxin, dobutamine) inotropic pharmacologically diverse compounds were assessed to identify if one particular cell type within the CMEF microtissue was responsible for the differences observed. Following exposure to the β1-adrenergic receptor antagonist, atenolol, a negative inotropic response was observed in both the CMEF and CMF microtissues, resulting in IC_50_ values of 0.94 μM; no effect was observed in CM, CME, or DMEF microtissues (Figure 4A). However the EGFR-1/EGFR-2 (Her2) inhibitor, lapatinib (Albanell and Gascon, 2005), produced a negative inotropic response in both CMEF and CME microtissues resulting in IC_50_ values of 0.86 and 1 μM respectively; no effect was observed in CM, CMF, and DMEF microtissues (Figure 4B).

Following exposure to the known *in vivo* positive inotropes, dobutamine, and digoxin, a positive inotropic response was only observed in the CMEF microtissue resulting in EC_50_ values of 0.3 and 3.54 μM, respectively, the converse response was observed in CM, CME, and CMF microtissues, resulting in IC_50_ values of 0.02, 0.01, and 0.001 μM respectively following dobutamine and 0.7, 0.9, and 0.3 μM, respectively following digoxin (Figs. 4C and D). DMEF microtissues displayed no response to either positive inotrope. These results highlight the requirement of both endothelial and fibroblast cells of cardiac origin to be present for the enhanced pharmacological response to be observed. To confirm that the enhanced pharmacological response of the multicellular microtissue was not restricted to microtissues composed of hESc-derived CMs we also utilized iPSc-derived CMs. Two microtissues containing hiPS-CMs were prepared; hiPS-CMs in combination with hCMECs and hCF (hiPS CMEF) and hiPS-CMs alone (hiPS CM). Following exposure to the known *in vivo* positive inotrope, dobutamine, a positive inotropic response was again only observed in the hiPS CMEF microtissue, resulting in an EC_50_ value of 0.39 μM in hiPS CMEF; the converse response was observed in the hiPS CM microtissue, resulting in an IC_50_ value of 0.1 μM (Figure 4E). These results indicate the suitability of both hESC-CM and hIPC-CM microtissues to detect changes in cardiac contractility.

**FIG. 5:**
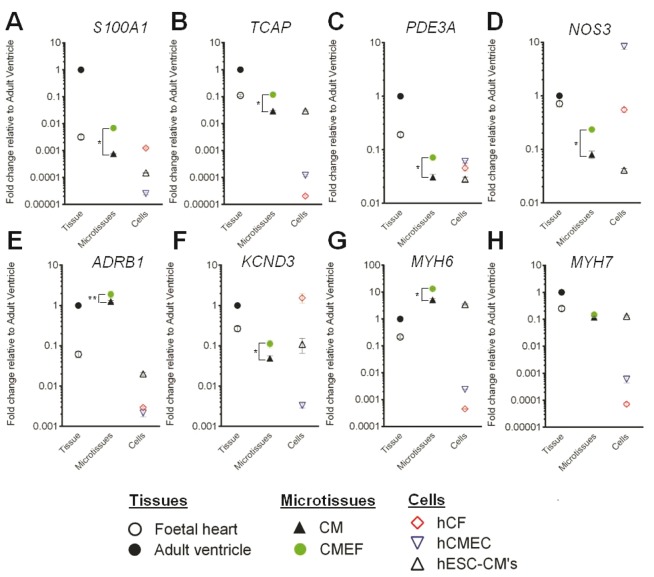
Gene expression upregulation in cardiac microtissues. Fold change of **(A)** *S100A1*, **(B)** *TCAP*, **(C)** *PDE3A*, **(D)** *NOS3*, **(E)** *ADRB1*, **(F)** *KCND3*, **(G)** *MYH6*, and **(H)** *MYH7* mRNA expression in foetal heart tissue, cardiac microtissues, and individual cardiac cells relative to adult heart tissue (n = 3, ± SD) was determined by qRT-PCR. *P-value < .01, **P-value < .05.

In order to provide further mechanistic insight into possible explanations for these enhanced physiologically relevant responses in CMEF microtissues, gene expression analysis of a panel of genes involved in microtubule and sarcomere assembly (*S100A1*; S100 calcium-binding protein A1, *TCAP*; titin-cap), cardiovascular function (*ADRB1*; β1-adrenergic receptor, *PDE3A*; phosphodiesterase 3, *KCND3*; potassium voltage-gated channel Kv4.3), nitric oxide production (*NOS3*; nitric oxide synthase 3) and cardiomyocyte maturity (*MYH6*; myosin heavy chain 6, *MYH7*; myosin heavy chain 7) were evaluated. When compared with the expression of these genes in monolayer hESC-CMs and CM microtissues, CMEF microtissues showed increased expression of *S100A1, TCAP, PDE3A, NOS3 and KCND3* (*P* value < .01) with fold changes of 0.007, 0.12, 0.07, 0.23, and 0.1 in CMEF microtissues and 0.0007, 0.03, 0.03, 0.08, and 0.05 in CM microtissues compared to adult ventricular tissue, respectively (Figs. 5A–D and F). These expression levels are comparable to those observed in foetal cardiac tissue. Whereas, *ADRB1* expression was similar to adult ventricle tissue in both CMEF and CM microtissues with fold changes of 1.9 and 1.3 (*P* value < .05), respectively, this is compared with a fold change of 0.02 in monolayer hESC-CMs (Figure 5E). *MYH7*, a predominantly adult myosin chain, was expressed at equal levels in CMEF and CM microtissues with a fold change of 0.15 relative to adult ventricle tissue, an expression level comparable to foetal heart and hESC-CM monolayer. *MYH6*, predominantly a foetal myosin chain, was highly expressed in CMEF and CM microtissues, 13- and 5–fold, respectively (*P* value < .01), compared with adult ventricle (Figs. 5G and H).

### S100A1 Regulates Contractile Maturity

**FIG. 6:**
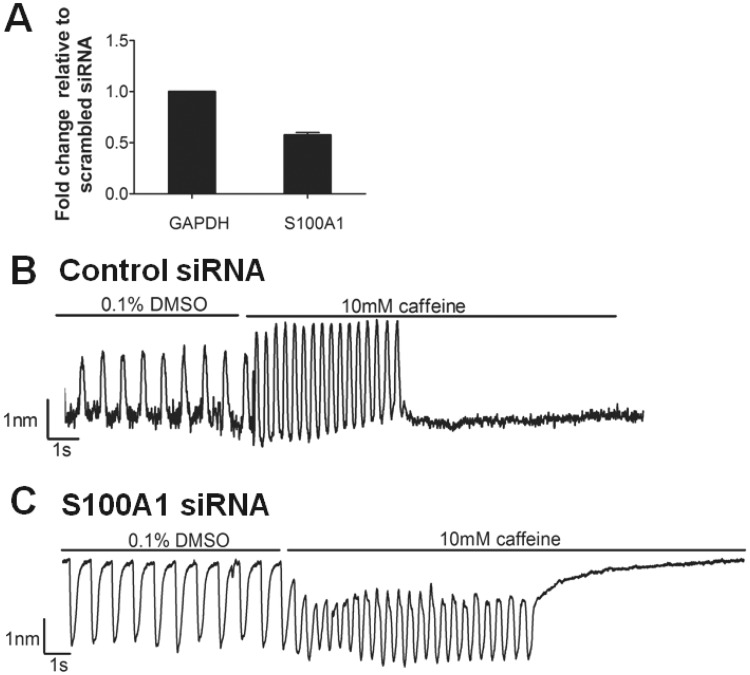
S100A1 regulates contraction in response to caffeine. CMEF microtissues were formed and incubated for 14 days prior to transfection with 10 nM nonsilencing siRNA (−ve control) and 10 nM S100A1 siRNA. **A,** RNA was extracted from microtissues and expression of *GAPDH* and *S100A1* mRNA analysed by qRT-PCR (n = 3, ± SD). Contractile response recorded using the IonOptix cell geometry system under vehicle control (0.1% v/v DMSO) and 10 mM caffeine in **(B)** nonsilencing siRNA control CMEF microtissue and **(C)** S100A1 siRNA transfected CMEF microtissue (results from 1 experiment representative of 3 separate experiments).

S100A1, a cardiac Ca^2+ ^regulator (Maco *et al.*, 2001), showed enhanced upregulation specifically within CMEF microtissues (Figure 5A). Considering the different caffeine-ICR responses of the CMEF microtissues we hypothesized that S100A1 may have a role in the contractile maturity of the CMEF microtissues. To confirm this, short interfering RNA (siRNA)-mediated knockdown of S100A1 was performed in CMEF microtissues and its effect on caffeine response studied. Partial knockdown of *S100A1 mRNA* expression by 42% ± 2.58 (mean ± SD) (Figure 6A), resulted in a complete loss of the transient increase in CMEF contraction amplitude following caffeine exposure (Figs. 6B and C).

## DISCUSSION

The human heart is composed of CMs, fibroblasts and endothelial cells, which facilitate normal cardiac development and physiological cardiac function (Brutsaert, 2003). Here we have shown that adult cardiac nonmyocyte cells can promote contractile maturity of embryonic stem cell derived CMs *in vitro* by co-culturing as a 3D microtissue to better recapitulate the *in vivo* cellular physiology and pharmacological response.

The advent of stem-cell derived human CMs offers the potential for more physiologically relevant preclinical safety testing for drug-induced cardiotoxicity and disease understanding. However, it has been widely reported that hESC-CMs structurally resemble embryonic CMs and lack the complex communications required for efficient excitation-contraction coupling (Kehat *et al.*, 2003). In the adult CM, isoproterenol, a β_1_-adrenergic receptor agonist, displays positive inotropy (increase in contraction amplitude), and positive chronotropy (increase in contraction rate). hESC-CMs elicit positive chronotropy but no positive inotropy suggesting hESC-CMs have some degree of β_1_-adrenergic receptor expression, but again highlighting the immaturity of this cell model (Robertson *et al.*, 2013). Here we have demonstrated the relatively low level β_1_-adrenergic receptor expression found in hESC-CMs can be increased using three dimensional cell culturing (Figure 5E).

The enhanced *in vitro* to *in vivo* pharmacological correlation observed in the CMEF microtissues compared with the CM microtissues was evident across a range of different negative inotropic drugs targeting a diverse set of targets (Table 1), eg, the β_1_-adrenergic receptor (atenolol) and EGFR-1/EGFR-2 receptor (lapatinib) and positive inotropic drugs targeting the β_1_-adrenergic receptor and Na/K+ ATPase (digoxin). The ability of atenolol to inhibit contraction in the absence of exogenous catecholamines can be potentially explained by either the inverse agonist properties of β-adrenoceptor antagonists (Varma *et al.*, 1999) or endogenous secretion of catecholamines by the microtissue (Sorriento *et al.*, 2012). The positive pharmacological correlation evident in the CMEF was restricted to cardiac endothelial cells and fibroblasts, as substitution with dermal equivalents of microvascular endothelial cells and fibroblasts in the microtissue did not reconstitute the positive correlation. This difference between the effect of CMEF microtissues compared with CM microtissues was not restricted to the use of hESc-derived CMs as a similar effect on contractility with dobutamine was observed using iPSc-derived CMs (Figure 4E).

This positive pharmacological effect was not due to differences in cellular architecture within the microtissues as the dermal nonmyocyte cells and cardiac nonmyocyte cells formed multicellular microtissues with a similar structure. However, differences in gene expression between the different multicellular microtissues suggest that the cardiac derived nonmyocytes are capable of inducing a transcriptional program within the CMs that results in pharmacological maturity. Analysis of gene expression in the different cardiac microtissues revealed that the Ca^2+ ^binding protein *S100A1* was upregulated in the CMEF microtissue relative to the CM microtissue (Figure 5A). The importance of this gene upregulation was confirmed using a partial siRNA-mediated knockdown CMEF model which highlighted a loss of inotropic response to a short timescale exposure of the Ca^2+ ^mobilizing agent caffeine. Studies have previously highlighted a correlation between S100A1 protein expression and inotropy response (Bennett *et al.*, 2014). Furthermore, adenoviral mediated over-expression of S100A1 improved β_1_ adrenergic mediated contractile reserve in CMs from failing human hearts, suggesting that this protein may play multiple roles in regulating cardiomyocyte physiology (Brinks *et al.*, 2011).

In adult CM, Ca^2+ ^induced Ca^2+ ^release (CICR) from the sarcoplasmic reticulum contributes almost 70% of the total Ca^2+ ^release. In contrast, immature CMs demonstrate Ca^2+ ^transients that are smaller and slower (Robertson *et al.*, 2013), with most cation influx of Ca^2+ ^through the cell membrane (Liu *et al.*, 2007). This results in abnormal diffusion of Ca^2+ ^into the cell and reduces the synchrony in contraction necessary for large force generation. There is also consensus that intracellular Ca^2+ ^stores are smaller than in adult CM and less reliant upon ryanodine receptors for Ca^2+ ^release. Ca^2+ ^handling and response to compounds that modify Ca^2+ ^handling varies greatly between immature and mature CMs. The differences in the amplitude of the Ca^2+ ^transient between CMEF and CM microtissues, that were not evident for the edge transient (Figs. 3G and H), could represent a difference in the excitation–contraction coupling process between the two microtissues, potentially due to differences in Ca^2+ ^handling and storage. Indeed, Ca^2+ ^currents have been recorded in both endothelial cells (Tran *et al.*, 2000) and cardiac fibroblasts (Li *et al.*, 2009), therefore Ca^2+ ^transients in these cell types could contribute to the increase in Ca^2+ ^transient amplitude in the CMEF microtissue. The increased Ca^2+ ^transient amplitude, response to Ca^2+ ^dyshomeostatic compounds (for example levosimenden and caffeine) and increased expression of the Ca^2+ ^handling protein S100A1 suggests a three dimensional co-culture with adult cardiac nonmyocytes can improve the immature Ca^2+ ^handling state of hESC-CM’s.

Together these findings show the promotion of maturity is very much multi-parameter dependent. However, it should be noted that despite achieving a functionally relevant degree of maturity, this *in vitro* myocardial model still lacks some of the characteristics of fully matured myocardial tissue. Additional studies to assess structural maturity such as T-tubule formation would provide further insight. Major morphological changes occur during prenatal to postnatal cardiac development as CMs adapt to changes in hemodynamic forces (Zimmermann, 2013). Adult CMs are large and cylindrical, approximately 150 μm in length. Despite the CMEF microtissue displaying improved maturity with regard to contractile function, the multicellular microtissues have a diameter of 200 μm, this probably reflects the lack of hemodynamic load during microtissue formation and maturation. Recent approaches to model tensile forces during microtissue development have utilized a microfabricated platform (Thavandiran *et al.*, 2013) and fibrin-based engineered heart tissue between 2 hollow elastic silicone posts (Hirt *et al.*, 2012). These engineered platforms promote cardiac maturation-associated gene expression similar to our study. However, we have provided a pharmacological interrogation of functional maturity in a scaffold-free multicellular microtissue.

The lack of a readily available source of human adult CMs for translation medicine means these stem cell-derived CMs have become fundamental to *in vitro* cardiac research. Our previous data, and that of others, has shown that stem-cell derived CMs are able to analyse both functional (Pointon *et al.*, 2015) and structural cardiotoxicity (Clements *et al.*, 2015; Pointon *et al.*, 2013). By improving the *in vitro* culture of these CMs to better recapitulate the *in vivo* cellular microenvironment within the myocardium, we have developed a novel model with enhanced pharmacological relevance. Importantly, this microtissue model can be utilized for analysis of functional and potentially structural cardiotoxicity, is amenable to high-throughput production and has the potential to highly impact cardiac research and analysis of drug-induced cardiovascular toxicity.

## SUPPLEMENTARY DATA

Supplementary data are available online at http://toxsci.oxfordjournals.org/.

Supplementary Data
