# Supplementary material for: Cardiac Non-myocyte Cells Show Enhanced Pharmacological Function Suggestive of Contractile Maturity in Stem Cell Derived Cardiomyocyte Microtissues
Source: Toxicol Sci. 2016 Apr 28;152(1):99–112. doi: 10.1093/toxsci/kfw069 (PMC4922542; doi:10.1093/toxsci/kfw069)
Supplement: Supplementary Data [file supp_152_1_99__index.html]

Cardiac Non-myocyte Cells Show Enhanced Pharmacological Function Suggestive of Contractile Maturity in Stem Cell Derived Cardiomyocyte Microtissues — Cardiac Non-myocyte Cells Show Enhanced Pharmacological Function Suggestive of Contractile Maturity in Stem Cell Derived Cardiomyocyte Microtissues — Supplementary Data 

# Cardiac Non-myocyte Cells Show Enhanced Pharmacological Function Suggestive of Contractile Maturity in Stem Cell Derived Cardiomyocyte Microtissues

## Supplementary Data

files

- Supplementary Data - zip file
