# Supplementary material for: Cardiac Non-myocyte Cells Show Enhanced Pharmacological Function Suggestive of Contractile Maturity in Stem Cell Derived Cardiomyocyte Microtissues
Source: Toxicol Sci. 2016 Apr 28;152(1):99–112. doi: 10.1093/toxsci/kfw069 (PMC4922542; doi:10.1093/toxsci/kfw069)
Supplement: Supplementary Data [file supp_kfw069_suppl_data.zip › toxsci-15-0788-File009.docx]

**Supplementary Table 1.**

Table 1. TaqMan® Probes

| **Gene** | **Gene Name** | **Primer Id** | **Catalogue Number** | **Supplier** |
| --- | --- | --- | --- | --- |
| S100A1 | S100 calcium binding protein A1 | Hs00984741_m1 | 4331182 | Life Technologies™ (Paisley, U.K.) |
| TCAP | Titin-cap | Hs00985784_g1 | 4331182 | Life Technologies™ (Paisley, U.K.) |
| PDE3A | phosphodiesterase 3A, cGMP-inhibited | Hs01012698_m1 | 4331182 | Life Technologies™ (Paisley, U.K.) |
| NOS3 | nitric oxide synthase 3 (endothelial cell) | Hs01574659_m1 | 4331182 | Life Technologies™ (Paisley, U.K.) |
| ADRB1 | adrenoceptor beta 1 | Hs02330048_s1 | 4331182 | Life Technologies™ (Paisley, U.K.) |
| KCND3 | potassium voltage-gated channel, Shal-related subfamily, member 3 | Hs00542597_m1 | 4331182 | Life Technologies™ (Paisley, U.K.) |
| MYH6 | myosin, heavy chain 6, cardiac muscle, alpha | Hs01101425_m1 | 4331182 | Life Technologies™ (Paisley, U.K.) |
| MYH7 | myosin, heavy chain 7, cardiac muscle, beta | Hs01110632_m1 | 4331182 | Life Technologies™ (Paisley, U.K.) |
| GAPDH | glyceraldehyde-3-phosphate dehydrogenase | Hs02758991_g1 | 4331182 | Life Technologies™ (Paisley, U.K.) |
